# Understanding the Mechanisms of Main Bronchial Compression in Patients with Intracardiac Anomalies

**DOI:** 10.1016/j.atssr.2024.03.008

**Published:** 2024-04-02

**Authors:** Makoto Ando, Takako Nishino

**Affiliations:** 1 Department of Pediatric Cardiovascular Surgery, Kanazawa Medical University, Ishikawaken, Japan

## Abstract

**Background:**

The study focuses on vascular compression of the main bronchus in the aortopulmonary space, examining potential contributors within the same axial plane. Its goal is to uncover mechanisms of bronchial compression in patients with intracardiac anomalies and review surgical outcomes, aiming to enhance future results.

**Methods:**

The morphology and topology of structures within the axial plane of the aortopulmonary space were objectively analyzed, including the sternum, ascending aorta, heart, pulmonary artery, descending aorta, and other relevant elements. Identified deviations from the normal configuration were systematically identified. Operative procedures included mobilizing and removing the compressing vessel, followed by suspending the airway wall to a rigid prosthesis (external stenting), vertebra, or ascending aorta.

**Results:**

Computed tomography revealed potential factors contributing to bronchial stenosis, including anteriorly deviated descending aorta (20 patients), dilated pulmonary artery (6), cardiomegaly (12), flat chest (7), funnel chest (3), posteriorly deviated ascending aorta after arterial switch operation (3), low aortic arch (3), and aberrant subclavian artery (2). Kaplan-Meier analysis demonstrated operative survival rates of 96% at 1 year, 87% at 5 years, and 80% at 8-15 years. Ten-year follow-up computed tomography after external stenting procedure revealed the narrowest diameter of the stented bronchus as 94.4% of the reference.

**Conclusions:**

Consistent long-term airway patency was observed post-surgery. While the pulmonary artery and descending aorta exert direct compressive effects in most cases, various other potential mechanisms may contribute to bronchial compression. Identifying and addressing these factors through a multidisciplinary approach is crucial for sustaining bronchial patency and preventing complications.

**In Short**

▪ Treatment options for vascular compression syndrome of the main bronchus include posterior translocation of the main bronchus and the descending aorta, achieved through techniques like aortopexy or posterior bronchopexy.
▪ Factors contributing to main bronchial compression include anteriorly displaced descending aorta, pulmonary arterial dilation, cardiomegaly, osseous chest deformities like pectus excavatum, and anatomical shifts post-surgery.
▪ A multidisciplinary approach combining surgical interventions like aortopexy with pharmacologic therapy for cardiomegaly is crucial for successful long-term management of main bronchial compression.

Airway obstruction from vascular compression syndrome (VCS), often linked to cardiovascular abnormalities, can lead to respiratory distress. The main bronchus (MB), susceptible to topologic changes due to abnormal hemodynamics or surgery, can be compressed by the dilated pulmonary artery (PA)^1^ or displaced descending aorta (DA).^2^ However, any other deviation from the normal structures within the aortopulmonary space (APS) plane may narrow the APS, causing MB compression. A comprehensive understanding of this is crucial for resolving MB obstruction, an area not explored in prior studies. This study aims to detail VCS mechanisms in patients with intracardiac anomalies, reviewing surgical outcomes, with the future goal of improvement.

## Patients and Methods

This study obtained approval from Kanazawa Medical University's institutional review board, with waived written informed consent.

### Demographics

Since 2008, 34 patients with intracardiac anomalies were diagnosed with the VCS of the MB and subsequently underwent surgical intervention. The mechanisms of VCS were quantitatively analyzed using sagittal plane assessments involving the APS. The median follow-up duration was 4.5 (maximum, 15.2) years.

### Analysis of the Computed Tomography Scan

**Figure 1:**
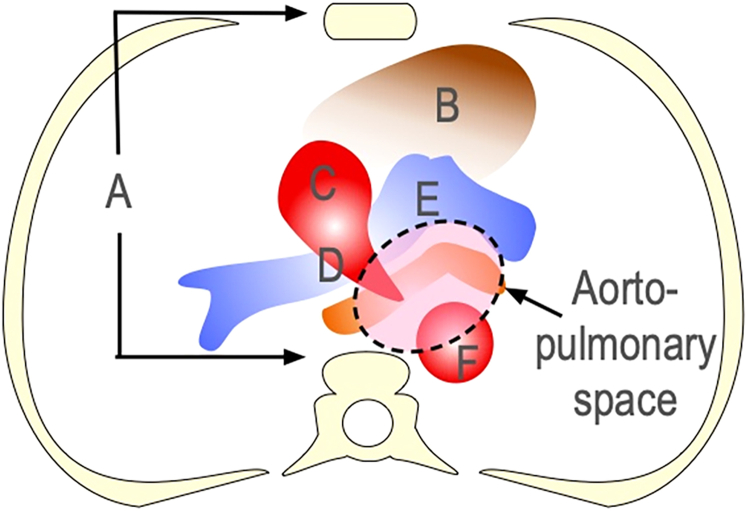
Structures on the axial computed tomography image at the level of the aortopulmonary space. Sternum-vertebra distance (A), heart (B), ascending aorta (C), aortic arch (D), pulmonary artery (E), descending aorta (F), and aberrant subclavian artery.

Examined structures in preoperative computed tomography (CT) included sternum, heart, ascending aorta (AA), aortic arch, PA, DA, and aberrant subclavian artery (ASCA) at the APS level (Figure 1). Morphologic changes were identified through specific methods. Haller index (ribcage inside distance divided by sternum-vertebrae distance) >2.5 indicated chest narrowing or presence of funnel chest.^3^ Anterior deviation of the DA (>50% in front of the vertebra), short aortic arch causing left MB compression, and dilated AA or PA (ratio > 0.4 m) were also investigated.

### Other Analysis

To evaluate long-term MB patency after external stenting (ES), >10-year follow-up CT scans were performed on 6 patients. Measurements of the narrowest stent segment diameter and the reference diameter proximal to the stent were taken on coronal and sagittal planes. Kaplan-Meier survival analysis was used for actuarial survival calculation in surgical patients. Statistical analyses were conducted using JMP software for Windows, version 13.0 (SAS Institute Inc).

## Results

The symptomatic reasons for the operation included dependence on mechanical ventilation in 18 patients, positive pressure breathing in 10, recurrence of pneumonia in 4, and episodes of life-threatening anoxic spells in 2. The affected airway was the left MB in 29 cases and the right in 5. Additionally, 3 patients had the trachea affected. There were 14 female and 20 male patients, with a median age of 6.5 months (range, 1.8-77.1 months). Intracardiac anomalies included coarctation/interruption complex in 11, hypoplastic left heart syndrome in 5, absent pulmonary valve in 3, atrial septal defect in 3, single ventricle in 3, transposition of the great arteries in 3, ventricular septal defect in 2, and 1 each case of tetralogy of Fallot, mitral stenosis, total anomalous pulmonary venous connection, and truncus arteriosus. Aortic arch repair had been previously performed in 18 cases.

The surgical procedures involved ES (31 patients), posterior bronchopexy (3), and fixation of the MB to AA (1). The surgical approach involved left thoracotomy in 27 cases, right thoracotomy in 5, and median sternotomy in 2. The stents used for ES were 16 mm in 9 cases, 14 mm in 11, and 12 mm in 11.

### Mechanisms of MB Compression

**Figure 2:**
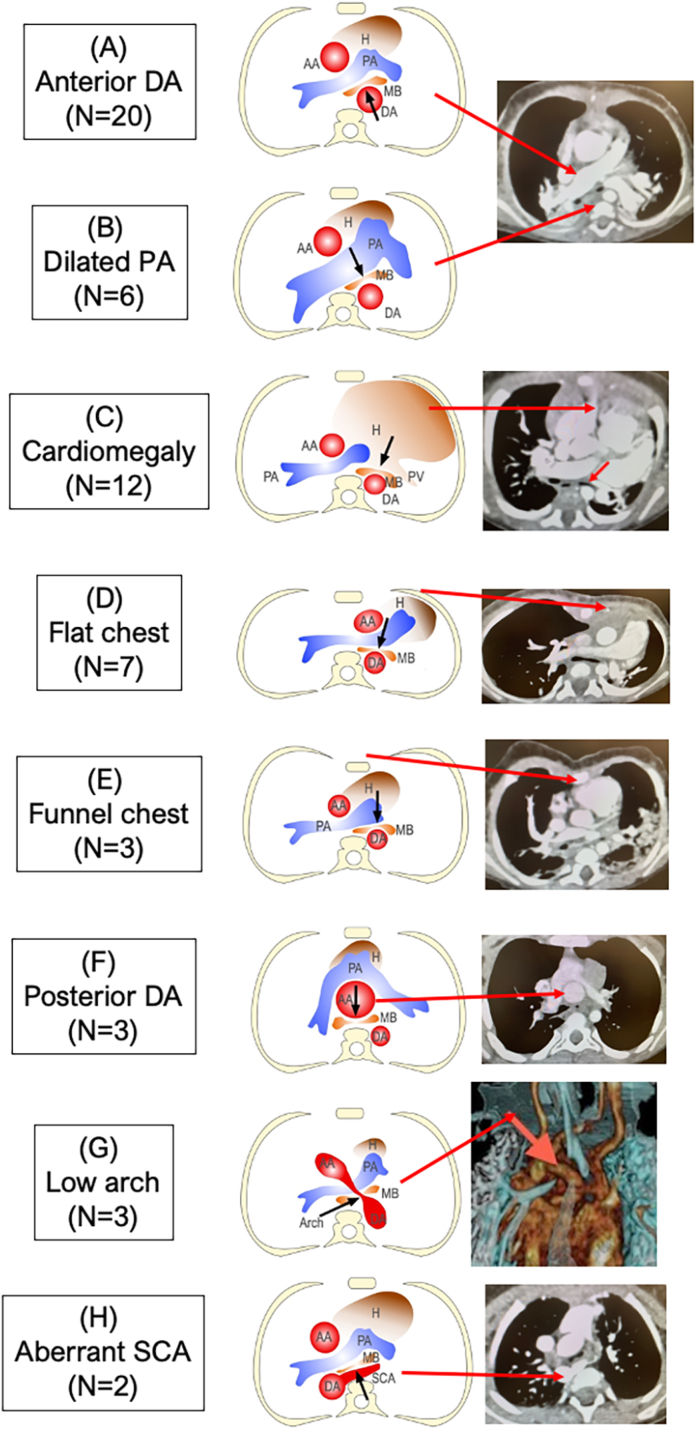
Elements potentially contributing to the main bronchial compression. (AA, ascending aorta; DA, descending aorta; H, heart; MB, main bronchus; PA, pulmonary artery; SCA, subclavian artery.)

Based on the CT scan, various potential contributing factors to the narrowing of the APS were identified (Supplemental Table 1). These include an anteriorly deviated DA (20 patients), a dilated PA (6), cardiomegaly (12), a flat chest (7), post-sternotomy funnel chest (3), posteriorly deviated and dilated AA after arterial switch operation (3), a low aortic arch (3), and ASCA (2) (Figure 2). In 19 patients, a single factor among the mentioned factors was involved. In the remaining patients, 2 or more factors were implicated: 2 factors in 9 patients, 3 factors in 5, and 4 factors in 1 (Table 1).

**Table 1:** Summary of Anatomical Abnormalities Observed on the Computed Tomography Scan and Potential Management Strategies for Each Condition

Abnormality	Number of Patients	Description	Management Strategy
Anteriorly displaced descending aorta	20	Common after arch reconstruction for arch obstruction	Posterior translocation of the aorta
Dilation of the pulmonary artery	6	Often linked to absent pulmonary valve syndrome and atrial septal defects	Anterior translocation of the pulmonary artery
Cardiomegaly	12	Can lead to extrinsic compression of the pulmonary vein	Pharmacologic treatments for cardiomegaly
Flat chest	7	Osseous narrowing of the sternum-to-vertebra distance	Addressing underlying condition
Funnel chest	3	Pectus excavatum, especially post-median sternotomy	Reexposing the chest and reapproximating the sternum
Posterior displacement of the ascending aorta	3	Common in patients with transposition of the great arteries following arterial switch	Modified external stenting technique
Low aortic arch	3	Aortic arch shortening, leading to a lowered arch	Complete mobilization of the thoracic aorta
Aberrant subclavian artery	2	Compressing the main bronchus from the posterior aspect	Removal of the aberrant artery

### Surgical Results

Twenty-nine patients were successfully weaned off the ventilator during their hospitalization, with a median intubation period of 3 days (range, 1-94 days). There were 4 mortality cases: 2 in-hospital due to aortic perforation, and 2 after discharge from sudden death. Five patients required stent removal at a median duration of 1.7 years (range, 1.3-6.6 years). The reasons included stent perforation to the DA in 2, stent infection in 1, stent perforation to the airway in 1, and obstruction of the PA in 1. Kaplan-Meier survival analysis revealed a 1-year survival rate of 96.2% ± 3.8%, a 5-year survival rate of 87.2% ± 6.9%, and a 79.9% ± 9.4% survival rate at 8.1 years postoperation. A follow-up CT scan conducted >10 years after the operation revealed the narrowest diameter of the airway per reference as 94.4% (with a minimum of 90.2%).

## Comment

### OPERATIONS for MB Compression

**Figure 3:**
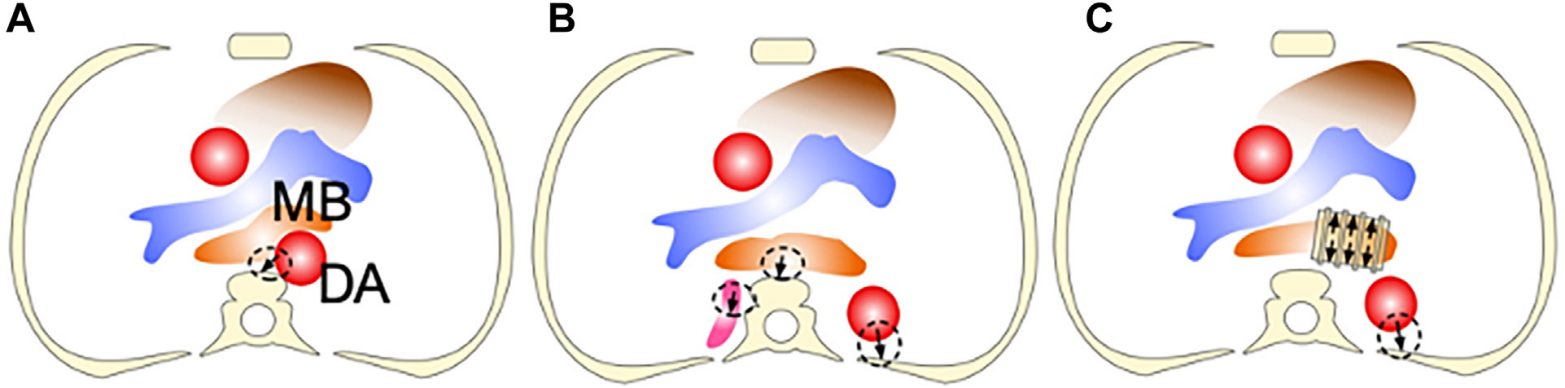
Technical scheme of (A) aortopexy, (B) posterior bronchopexy, and (C) external stenting. Arrows show retraction directions. (DA, descending aorta; MB, main bronchus.)

VCS, common in children with vulnerable airways, is often linked to an anteriorly deviated DA, especially after aortic arch surgery. Hence, a viable treatment option for VCS of the MB involves posterior translocation of both the MB and the DA. Aortopexy, the commonly used method, achieves this by suturing the DA to the vertebra (Figure 3A). Technically, a stitch is strategically placed to induce a rotational movement of the DA,^4^ facilitating the retraction of the MB towards the vertebra. Due to this technical intricacy, the effectiveness may vary depending on the alignment of the vertebrae, the DA, and the MB. Therefore, we favor the posterior bronchopexy technique, akin to aortopexy but distinctively involving separate retraction of the MB and DA, which may prove more efficient in retracting the MB. In this technique, the DA is secured to the ribs, requiring esophageal mobilization and relocation (Figure 3B). Both methods have limitations. Unlike aortopexy for the trachea, where the contralateral airway side is stabilized by fibrous tissue, this approach may be less effective for MB embedded in soft and mobile lung tissue. Moreover, prolonged compression can lead to irreversible airway structural abnormalities. An alternative approach, ES (Figure 3C) involves 360° support with a rigid prosthesis,^5^ demonstrating reliability and durability in expanding the airway. ES limitations include potential pressure on adjacent structures, raising concerns about perforation. To mitigate this, we now include the placement of an expanded polytetrafluoroethylene sheet that completely encircles the stent, effectively eliminating the risk of perforation.^6^ In our current practice, we initially use posterior bronchopexy, seamlessly transitioning to ES if MB narrowing persists.

### Factors Contributing to MB Compression

As mentioned before, the following 8 elements on the APS horizontal plane may contribute to MB VCS (Figure 2).

A. Anteriorly displaced DA: This is common in cases of arch obstruction, including interrupted aortic arch and coarctation of the aorta, after surgical repair. This compression is likely relieved by posterior translocation of the DA.
B. Dilation of the PA: This condition, often linked to absent pulmonary valve syndrome and atrial septal defects,^7^ has established surgical options. Although PA plication is viable, a more reliable approach is anterior translocation of the PA to the AA, effectively relieving anterior MB compression.^8^
C. Cardiomegaly: Cardiomegaly can lead to extrinsic compression of the pulmonary vein and may contribute to the onset of VCS of the MB. This can result from left atrium enlargement or posterior shift of the PA, compressing the posterior MB.^9^ Pharmacologic treatments for cardiomegaly are crucial in alleviating airway obstruction symptoms or maintaining MB patency after surgical interventions.
D. Flat chest: Osseous narrowing of the sternum-to-vertebra distance, often seen in conditions like pectus excavatum or a flat chest with a large Haller index, can contribute to compression.^10^ In adults, a typical Haller index is around 2.5, but deformities like pectus excavatum elevate it. Congenitally flat chests cause a leftward shift in cardiovascular structures, resulting in mutual compression of the AA, PA, MB, and DA.
E. Funnel chest: Pectus excavatum can occur after median sternotomy, especially in fragile pediatric sternums. Unlike isolated pectus, addressing this condition is relatively straightforward by reexposing the chest and reapproximating the sternum. This can be done during chest reopening at cardiac reoperation or as part of a planned staged repair.
F. Posterior displacement of the AA: In patients with transposition of the great arteries after arterial switch, AA dilation is common. Challenges in AA retraction or size reduction arise due to the anteriorly located pulmonary artery, limiting surgical options. To address this, we use a modified ES technique, placing half of the stent on the MB's anterior aspect against the vertebra, using the vertebra as a posterior splint.^6^ Two patients have successfully undergone this procedure, but concerns about aortic perforation persist, requiring mandatory stent isolation with a polytetrafluoroethylene sheet.
G. Low aortic arch: Corrective surgery often involves aortic arch shortening, leading to a lowered arch. This requires complete mobilization of the thoracic aorta from the arch to the diaphragmatic aorta just above the diaphragm, ensuring ample space for performing posterior bronchopexy or an ES in the conventional manner. In our experience, surgically elongating the aortic arch using a patch or interposing graft is unnecessary.
H. ASCA: In situations where the ASCA serves as the posterior vessel compressing the MB, the removal of this artery becomes imperative.

### Conclusions

PA and DA directly compress the MB, but other factors may also contribute. Identifying detailed mechanisms is crucial for considering additional treatments like pharmacologic therapy for cardiomegaly or surgical correction of funnel chest. A multidisciplinary approach is essential for successful repair, ensuring long-term MB patency and preventing complications.
