# Supplementary material for: Understanding the Mechanisms of Main Bronchial Compression in Patients with Intracardiac Anomalies
Source: Ann Thorac Surg Short Rep. 2024 Apr 2;2(3):369–73. doi: 10.1016/j.atssr.2024.03.008 (PMC11708380; doi:10.1016/j.atssr.2024.03.008)
Supplement: Supplemental Table [file mmc1.docx]

Supplemental table:Cardiac diagnoses and factors contributing to bronchial compression

| Case | Cardiac diagnosis | Anterior DA | Cardiomegaly | Flat chest | Dilated PA | Dilated AA | Pectus | Low arch | ASCA |
| --- | --- | --- | --- | --- | --- | --- | --- | --- | --- |
| 1 | Absent PA valve |  |  | **◯** |  |  |  |  |  |
| 2 | Absent PA valve |  |  |  | **◯** |  |  |  |  |
| 3 | Absent PA valve |  |  |  | **◯** |  |  |  |  |
| 4 | ASD+/-PAPVC | **◯** | **◯** |  | **◯** |  |  |  |  |
| 5 | ASD+/-PAPVC | **◯** | **◯** |  |  |  |  |  |  |
| 6 | ASD+/-PAPVC | **◯** |  |  | **◯** |  |  |  |  |
| 7 | CoA/IAA complex | **◯** |  |  |  |  |  |  |  |
| 8 | CoA/IAA complex | **◯** |  |  |  |  |  |  |  |
| 9 | CoA/IAA complex | **◯** | **◯** | **◯** |  |  |  | **◯** |  |
| 10 | CoA/IAA complex | **◯** | **◯** |  |  |  |  | **◯** |  |
| 11 | CoA/IAA complex | **◯** | **◯** |  |  |  |  |  |  |
| 12 | CoA/IAA complex | **◯** |  |  |  |  | **◯** |  |  |
| 13 | CoA/IAA complex | **◯** |  |  |  |  |  |  |  |
| 14 | CoA/IAA complex | **◯** |  |  |  |  |  |  |  |
| 15 | CoA/IAA complex | **◯** |  |  |  |  |  |  |  |
| 16 | CoA/IAA complex | **◯** |  |  |  |  |  |  |  |
| 17 | CoA/IAA complex |  |  |  |  |  |  | **◯** |  |
| 18 | FSV | **◯** | **◯** |  |  |  |  |  |  |
| 19 | FSV | **◯** |  |  |  |  |  |  |  |
| 20 | FSV | **◯** | **◯** |  |  |  |  |  | **◯** |
| 21 | HLHS/variant | **◯** |  | **◯** |  |  |  |  |  |
| 22 | HLHS/variant | **◯** |  |  |  |  |  |  |  |
| 23 | HLHS/variant | **◯** |  |  |  |  |  |  |  |
| 24 | HLHS/variant |  | **◯** |  |  |  |  |  |  |
| 25 | HLHS/variant |  | **◯** |  |  |  |  |  |  |
| 26 | MSR |  | **◯** |  |  |  |  |  |  |
| 27 | TAPVC |  |  |  |  |  | **◯** |  |  |
| 28 | TGA |  |  | **◯** |  | **◯** |  |  | **◯** |
| 29 | TGA |  |  | **◯** |  | **◯** |  |  |  |
| 30 | TGA |  |  |  |  | **◯** |  |  |  |
| 31 | TOF |  |  |  | **◯** |  |  |  |  |
| 32 | Truncus | **◯** |  | **◯** | **◯** |  |  |  |  |
| 33 | VSD |  | **◯** | **◯** |  |  |  |  |  |
| 34 | VSD |  | **◯** |  |  |  | **◯** |  |  |

AA, Ascending aorta; ASCA, Aberrant subclavian artery; ASD, Atrial septal defect; CoA / IAA, Coarctation or interrupted aortic arch; DA, Descending aorta; FSV, Functionally single ventricle; HLHS, Hypoplastic left heart syndrome; MSR, Mitral stenosis or regurgitation; PA, Pulmonary artery; PAPVC, Partial anomalous pulmonary venous connection; TAPVC, Total anomalous pulmonary venous connection; TGA, Transposition of the great arteries; TOF, Tetralogy of Fallot; VSD, Ventricular septal defect
